# Social Network Analysis of Factors Influencing Green Building Development in China

**DOI:** 10.3390/ijerph15122684

**Published:** 2018-11-28

**Authors:** Ning Huang, Libiao Bai, Hailing Wang, Qiang Du, Long Shao, Jingtao Li

**Affiliations:** School of Economics and Management, Chang’an University, Middle Section of South Second Ring Road, Xi’an 710064, China; Ning_Huang@chd.edu.cn (N.H.); hailing711@163.com (H.W.); q.du@chd.edu.cn (Q.D.); long_shao@chd.edu.cn (L.S.); lijingtao@chd.edu.cn (J.L.)

**Keywords:** green buildings, influencing factor analysis, social network analysis, life cycle and stakeholders

## Abstract

Green buildings have been viewed as one of the most effective solutions to the negative environmental impacts of construction activities. For the sustainable development of the economy and the environment, many governments in the world have launched a variety of policies to encourage the development of green buildings. However, green targets achieved during the operational stage of green buildings are far below the expectations from the design stage. In addition, the development of green buildings is unevenly distributed in different cities. To help resolve these issues, this paper identifies 28 green building influencing factors from two perspectives, the life cycle and stakeholders. Then, a social network analysis is used to analyse their interactions and identify the critical factors. Our results show that government supervision, incremental cost, property management experience, and the awareness of environmental protection in green buildings are the critical influencing factors in promoting green building development. However, some factors related to contractors, designers and suppliers are not as important as perceived. Finally, some policy recommendations are proposed to promote green buildings in China.

## 1. Introduction

With the reform of the housing market in China, the construction industry has developed rapidly and become a pillar of the Chinese national economy [1]. However, the construction industry consumes more than 40% of the total global energy. At the same time, more than 40% of global greenhouse gases (GHGs), which exert significant impacts on the climate, are emitted by this industry, resulting in a series of problems for the sustainable development of society [2]. Thus, many scholars have conducted related research and found that green buildings could be used as a strategy to reduce the environmental impact of the construction industry [3,4,5]. Green building is a more resource-saving and environmentally friendly form of construction. It can maximize the conservation of resources, protect the environment, reduce pollution throughout the life cycle of construction and provide people with healthy and suitable spaces [6,7]. Some studies indicate that green buildings could contribute to 35% reduction in GHGs, 70% reduction in waste output, and 70% savings on water usage [8].

Due to the numerous sustainability benefits, many governments and other organizations have taken measures to promote the development of green buildings. The Chinese government introduced the concept of green buildings in 1995 and continuously improved relevant standards [9]. Green buildings should be land-saving energy-saving, water-saving, and material-saving; and improve indoor environmental quality, construction management and operational management. It was defined by the Ministry of Housing and Urban-rural Development (MOHURD) and enacted in 2014 [10]. In addition, the Chinese government has developed many programmes that encourage green buildings. For example, the Chinese government proposed developing 10 million new affordable green buildings every year in the next 10 years [11].

**Figure 1:**
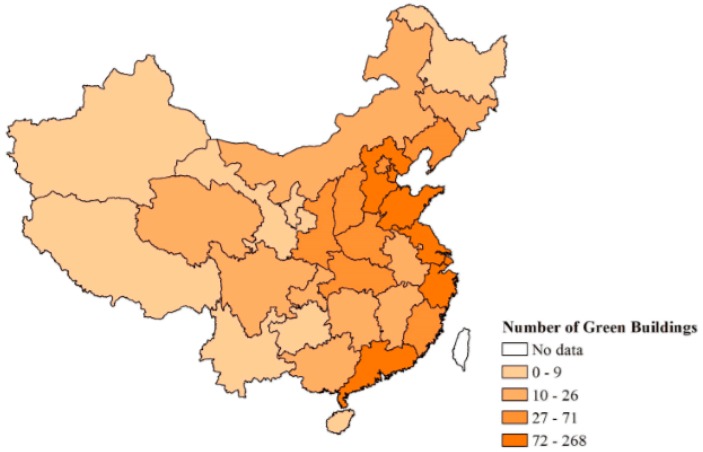
Distribution of green residential buildings in 2015.

As such, green buildings are booming and growing at an alarming rate every year in China, but there are two important issues that need to be addressed in their development. The first issue is that most green buildings are certificated only during the design stage. In China, due to the immature market mechanism, the development of green buildings mainly relies on government guidance, which includes green building technical specifications, green building evaluation standards, and the mandatory and incentive policies. Although the certification of green buildings requires application, approval and supervision, the evaluation of existing green buildings is limited to the design stage. This is different from the green building evaluation system in developed countries, such as the Leadership in Energy and Environmental Design (LEED) evaluation standard in the United States and Building Research Establishment Environmental Assessment Method (BREEAM) in the United Kingdom. These evaluation systems have specific evaluation criteria for each stage of the green building life cycle. According to information released by MOHURD, the amount of buildings obtained “Chinese Green Building Label” certification during 2008–2015 can been shown as Table 1. And the green buildings which were certified in the operational phase is much less than those in the design phase. For example, the number of residential green buildings in the design stage in 2013, 2014, and 2015 were 265, 431, 444, respectively, while the number of residential green buildings in the operation stage in 2013, 2014, and 2015 were 22, 17, 21, respectively [12]. The other issue is that the development of green buildings is unbalanced in different regions. Green buildings in China has developed for decades and has made great progress in quantity. However, these green buildings are mainly concentrated in the principal cities, for example, in 2015, 80% of green buildings is concentrated in 20% of cities according to information released by MOHURD [13], shown as Figure 1. In recent years, the gap has been alleviated but is still noticeable. This result is related to China’s unique regional development imbalance [14]. 

The identification of key factors is extremely important to promote the development of green buildings. In recent years, although the number of studies on green building factors has increased significantly, most studies are based solely on lifecycle or stakeholder perspectives and lack of research on the relationship between different influencing factors [15]. Both researchers and practitioners agree that green buildings are more complex and problematic because the construction industry is “extremely conservative” and subject to slow rates of change. Therefore, it is important to clearly identify the relationship among factors and find the important factors in the development of green buildings. To this end, in this paper, we first analyse all the influencing factors involved from the perspectives of the lifecycle and stakeholders. Then, the Social Network Analysis (SNA) method is employed to conduct a comprehensive analysis of the influencing factors and quantify their impact on green building performance. Finally, the relationship between different influencing factors is addressed, and the significant influencing factors are identified. The findings of this paper can guide stakeholders to make the correct decision, thereby furthering the development of the green building industry. To our knowledge, this paper fills the gap of previous research on green buildings that rarely measure the relationship between different influencing factors. Meanwhile, the analysis of influencing factors from the perspective of the lifecycle and stakeholders also provides a useful reference for future research.

The remainder of this paper is structured as follows: Section 2 reviews the influencing factors of green building literature; Section 3 describes the SNA and relevant research methodologies; Section 4 presents the results and indicators analysis; Section 5 shows the discussions about the research; and the conclusions, limitations of this study, and future research directions are presented in Section 6.

## 2. Literature Review

Global awareness of climate change, excessive energy consumption, waste of resources and environmental pollution in the construction industry are increasing; therefore, there is an increased demand for green buildings. In addition, research on green buildings has received significant attention in recent years [16]. Our research is mainly related to two research streams in the literature: First, this paper reviews the existing research on green building factors, including green building barriers and drivers. Second, this paper summarizes the research methods of green building factors and provides strong support for the selection of appropriate methods for this study. In what follows, we will review the papers in each research stream.

On the basis of an exhaustive literature review, it was found that existing green building research mainly focuses on barriers and driving factors. Zhang et al. argued that the financial factor was the biggest critical barrier, apart from weak enforcement of legislation and lack of motivation, that could be translated to green buildings objective in China [17]. Yau carried out a study in Hong Kong and stressed information asymmetry between different stakeholders around the environmental performance of green buildings as a crucial barrier [18]. In reality, clients may be not willing to purchase or pay more for green buildings due to a lack of knowledge about the operational benefits which discouraging the enthusiasm of developers [19]. Lam et al. argued that green equipment and materials in the construction stage are crucial for achieving green building targets [20]. The limitation of the scope and applicability of green equipment and materials are often considered an important barrier in reducing the efficiency of green buildings, which may force stakeholders to choose traditional building methods [21]. Shen et al. argued that the lack of a benchmarking system is another barrier for assessing and monitoring the performance of green construction. They advise contractors and suppliers should be engaged during the early stage of green building projects due to their knowledge of the environmental issues associated with construction activities, building materials and plants [22,23].

Driver factors also play an important role in the process of adopting green buildings. According to the development status of green buildings in China, Li et al. have studied and proposed some suggestions, including taking measures to enhance awareness of stakeholders, strengthening technological research and communication and establishing codes and regulations [24]. To assist in the promotion of green buildings, a life cycle approach should be considered during the assessment of relevant cost and impacts [21]. Furthermore, knowledge and understanding of sustainability by all parties, including policy makers, owners, designers, construction personnel and the public, need to be further enhanced. Bai et al. highlighted that mature green project management evaluation model play a significant in terms of both the costs and benefits associated with green buildings [25]. In many practical projects, the degree of support from senior management directly affects the adoption of green buildings [26]. Due to its higher population and building density and less available reusable energy per square metre of floor area, the development of sustainable building technologies that are applicable to various conditions in China is necessary [27]. Green construction demands the integration of sustainable technologies and interaction with other building components [28]. Practices such as “Soft landings” have been adopted in some countries and involve professionals after the completion of the building to ensure that the building actually works as anticipated. On the one hand, this practice creates a feedback loop for the project; on the other hand, it introduces challenges to contract management [29,30]. Based on a literature review, many studies have investigated the factors that promote the adoption of green buildings. However, most studies are based solely on lifecycle or stakeholder perspectives. Therefore, this study analyses all the influencing factors involved from the perspective of the lifecycle and stakeholders. This process can improve the comprehensiveness and accuracy of factor collection.

In terms of research methods, the past decade has witnessed a number of studies examining the factors of green building development. A questionnaire survey by Qi et al. given to construction contractors identified managerial concerns, government regulations and business size as the most important drivers for green practices [31]. Shi et al.’s survey on the adoption of green construction in China identified that economic factors were the most dominant barrier. In fact, control costs present the largest challenge to implementing green practices in China [26,32]. Berry et al.’s case study highlighted the exemplary leadership and spillover effects of niche events [33]. Li et al. employed the Tobit model and Cox proportional hazard model to investigate the drivers of green building development [12]. These studies investigated green building-related factors in different ways, but little attention was paid to the relationship between different influencing factors. In practice, the impact factors related to green buildings that are associated with various stakeholders are more complex than the factors related to traditional buildings. In addition, in the life cycle of green buildings, factors interact and form a network, implying that even a small variation in one factor might affect other factors in the network. Thus, the research processes on the relationship among green building influencing factors are extremely complex. SNA is an effective tool for researchers and practitioners to model organizational structure and analyse interactions among different individuals or groups, and this concept was introduced by Moreno in 1934 [34]. For example, Hagedoorn applied it to the analysis of strategic alliances [35]. Park et al. used it to filter sustainable technology from patent documents [36]. Ghali et al. used it to investigate the potential role of social connections and to simulate social connections [37]. In this paper, SNA is used to identify and quantify the interrelation among the different factors related to green buildings. With the aid of the SNA method, the mutual influence degree of each influencing factor is evaluated quantitatively, and the key factors restricting the development of green buildings are also determined. This information could also provide scientific suggestions for urban sustainable development.

## 3. Methodology

This paper uses a social network, which is joined by various stakeholders and related influence factors, to analyse the complex relationship in green buildings. Social network analysis is used to examine the existence and strength of the relationship between any two factors. And this theory focuses on the “structure and patterning” of these relationships and seeks to identify both their causes and effects [38,39]. In general, there are five steps of SNA [40] as follows:

### 3.1. Identification of the Stakeholders and Influencing Factors

The establishment of a social network is based on two parts: nodes and links. This part is to identify social network nodes. The objective is to find the stakeholders involved in the different stages of green buildings and identify their influencing factors. This research first involved a large amount of original collections of “who are the stakeholders” and “what are the influencing factors” in academic literature, publications by international organizations, and corporate reports. On the basis, the Delphi method can be used to screen out a comprehensive and reasonable list. The advantages of this identification method can be summarized as follows: (1) the collection of raw data is more complete and more scientific. (2) for most questions, experts can make educated responses based on their experience. The outcomes of this step will be a complete list of stakeholders and related influencing factors. All of the stakeholder-associated green building influencing factors will be numerically coded with S*F#, in which * implies the number of associated stakeholders and # is the influencing factor number related to this stakeholder. For example, S3F2 is the second influencing factor associated with the third stakeholder. In this part, the nodes of the influencing factor network are identified.

### 3.2. Determination of Influencing Factor Interrelations

This part defines the links in the influencing factor network that represent the relationship between each node. Following Steward’s work, the Design Structure Matrix method is used to build relations and dependencies among nodes in this step [41]. Based on Yang and Zou’s point of view [40], this research defines three relationships between each pair of influencing factors in the organizational structure:

(1) Dependent relationship: influencing factors are directly impacting each other;
(2) Independent relationship: there is no relationship between the two influencing factors;
(3) Interdependent relationship: there is no direct relationship between the two influencing factors, but there is an indirect connection between them across the network.

Previous research has mainly focused on a single factor in green buildings. The process included an evaluation of influencing factors and consequences and the likelihood of each factor affecting objectives. In this study, the objective is to define the relationship and the likelihood of an interaction between the influencing factors by the impact as opposed to by an analysis of individual factors. The method of developing an influencing factor structure matrix includes (1) a survey with key stakeholders and (2) workshops with relevant experts. The process should minimize bias effects of dominant individuals and group pressure for conformity. A certainty check between each pair of influencing factors should be conducted. Due to the limitation of reality, only key stakeholders are involved in the actual investigation process. The outcomes from this step are the defined links in the influencing factor network.

### 3.3. Visualization of the Influencing Factor Network

After the completion of the first two steps outlined in Section 3.1 and Section 3.2, an influencing factor network for green building can be developed. The network can be represented by a graph G (M, N), in which the influencing factors are mapped into M nodes and connected by N weighted arrows [42]. In the network, different shapes of the nodes represent influencing factors associated with different stakeholders, while different colours of the nodes represent different influencing factor categories. The arrows with values in the network are the interrelations among the influencing factors, of which the thicknesses indicate the degrees of influence (i.e., impact × likelihoods) of the interrelations.

### 3.4. Determining the Influencing Factor Network

Network measures, node/link measures, and partition measures are three useful types of measures for network analysis in the SNA model. These indicators can be used to decipher the structural configuration of the influencing factor relations.

#### 3.4.1. Network Measures

Network measures include density measures and cohesion measures that are usually used to describe the characteristics of an entire network.

(1) Density is defined as the proportion of actual ties present in a network to the maximum number of potential ties if every actor is connected with others [43]. Density can be calculated by Equation (1). Network density ranges between 0 and 1. The higher the density value, the more influence interrelations are in the network.
  (1) $$D=\frac{M}{n(n-1)}$$
  where *D* is the network density; *M* is the number of edges in the network; *n* is the number of nodes in the network; and *n* × (*n* − 1) is the potential maximum number of edges in the industry network.
(2) Cohesion is defined as network complexity based on the reachability of nodes. Specifically, cohesion indicates the distance and the number of ties to reach nodes in a network according to the shortest path [44]. Cohesion is measured by calculating how many paths of length 1 there are from each node to another node, as shown in Equation (2). The higher the cohesion, the closer the influencing factors are connected in the network.
  (2) $$l=\frac{\sum_{i\neq j}b_{ij}}{n(n-1)}$$
  where *n* is the number of nodes in the network and *b_ij_* is the shortest path length between node *i* and node *j*.

#### 3.4.2. Node/Link Measures

Node/link measures have four main indicators: degree of nodes, betweenness centrality, status centrality and brokerage. These indictors can be used to describe the immediate and/or mediate relationships among the individual actors in a network.

(1) The degree of nodes indicates the immediate connectivity characteristic of an influencing factor. In-degree and out-degree are two network measures that are adopted in this research. “In-degree” refers to incoming relations (impacted by) and “out-degree” to outcoming relations (impact to) [45]. The degree value indicates the links between influencing factor S*F# and its neighbours throughout the network. Each node degree can be acquired by calculating the weight sum of links as presented in Equations (3) and (4). The higher the in-degree value of one node is, the stronger the impact of the node received from others. The higher the out-degree value of one node is, the stronger the impact of the node to the others.
  (3) $$D_{i}^{in}=\sum_{j=1}^{n}x_{ji}/(n-1)$$
  (4) $$D_{i}^{out}=\sum_{j=1}^{n}x_{ij}/(n-1)$$
  where *D* is the network density; *M* is the number of edges in the network; *n* is the number of nodes in the network; and *n* × (*n* − 1) is the potential maximum number of edges in the industry network.
(2) Betweenness centrality provides an indication that the specific node/link is located between other pairs of nodes/links [46]. The node/link value of betweenness centrality shows the level of the impact passing through it, and the node is acting in a gatekeeper role. The calculation is based on the shortest path, as shown in Equations (2) and (5).
  (5) $$B_{i}=\frac{\sum_{j<k,i\neq j,i\neq k}g_{jk}(n_{i})/g_{jk}}{(n-1)\times (n-2)/2}$$
  where *B_i_* is the betweenness centrality of node *i*; *g_jk_*(*n_i_*) is the number of shortest paths between *j* and *k* through *i*; and *g_jk_* is the total number of shortest paths between *j* and *k*.
(3) State centrality is a type of node metric that analyses the relative impact of nodes in a network by measuring the number of direct neighbours (first degree nodes) and all other nodes in the network that are connected to the node under consideration by these direct neighbours. The in-status/out-status centrality denotes the extent to which a factor is affected by others or a factor can affect the others, respectively [47]. This study uses out-status centrality as the outcome measure. The higher the value of the out-status centrality is, the greater the impact of the factor on other factors.

### 3.5. Identification of the Critical Influencing Factors

The SNA model has been demonstrated as a useful tool for assessing factor interactions in green building projects by Yong and Zou (2016) [48]. Therefore, we use this model to identify the critical influencing factors and interrelations based on the results in the last step.

## 4. Results

### 4.1. Building the Influencing Factor Network

Twenty experts, including 8 construction management scholars and 12 industrial project managers were invited to join a Delphi panel, two-round Delphi survey was carried out for screening the identified green building influencing factors. In the first round, the background and purpose of this research were sent to the respondents by e-mail. And the respondents were asked to screen the collected influencing factors of green building and stated their reasons. In the second stage, all the data and information of the previous round was analyzed. And the results were provided to these experts so that they can rethink and judge the selected influencing factors. In total, 28 “green-related” influencing factors associated with 11 stakeholders were identified, as shown in Table 2.

**Figure 2:**
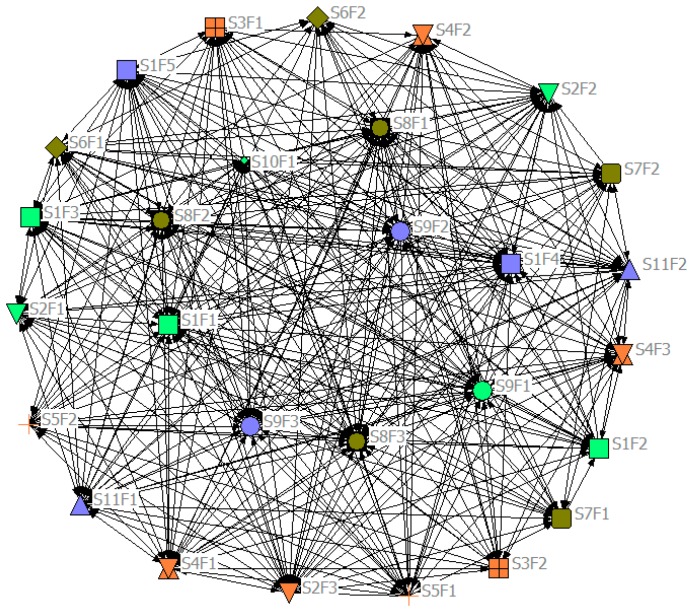
Stakeholder-associated influencing factor network.

In this paper, the relationship between each pair of influencing factors are identified by the approaches of questionnaire surveys and workshops with key stakeholders or relevant experts. The questionnaire survey was started in November 2017, after initial testing of a small number of respondents and corrected base on their comments, a questionnaire was issued to 300 respondents through the questionnaire survey software platform named “Questionnaire Star” (Changsha Raoxing Information Technology Co., Ltd., Changsha, China), and only those who have more than three years of experience in studying, producing or using Chinese green building products can be selected as respondents in order to ensure the reliability of survey results. Because the respondents are involved in different industries and their contributions are difficult to measure accurately, the determination of respondents’ weights is very complex. To simplify the complexity of the process, we use a uniform standard for the results of the survey and judge the relationship between each pair of factors based on the set pre-values. After this survey, nine experts of green buildings, made up of developers (two representative), designers (two representatives), contractors (two representatives) and academic (three representatives), were invited to conduct workshops, all of them have more than ten years of work experience and have an extremely profound understanding of green construction. Therefore, the survey results of these experts with a certain scientificity and accuracy. Based on the results of questionnaire surveys and workshops, the green building influencing factor network G (11, 478) could be established by building and analyzing the matrix of relationships between different factors, shown as Figure 2. In Figure 2, the node shapes represent the stakeholder-associated influencing factors, while the colours indicate the stakeholders. In the network, the critical influencing factors are located in a central position with more links than others in the network. Figure 2 shows that round nodes occupy the central location of the network map, which implies that government has more influence on other stakeholders during the process of green building implementation. The formation of the network is based on the relationship of all factors, indicating that the research processes are extremely complex. In the complicated system, most of the green-related influencing factors and interrelationships are mainly concentrated in developers, contractors, end users and governments. Their interactions account for the majority of the existing links.

### 4.2. Results of Network Analysis

The indicators in Section 3.4 are used to quantitatively describe the configuration of the influencing factor network in green buildings. The network density is equal to 0.6323, the factors are connected via an average of 6.188 walks showing that the network is relatively dense compared to density value in [40], and the nodes are near each other. The cohesion value of the network, introduced in Equation (2), is equal to 0.669, which is higher than the network density. This result implies that the structure of the influencing factor network is more complex when considering the node approachability in the whole network.

**Figure 3:**
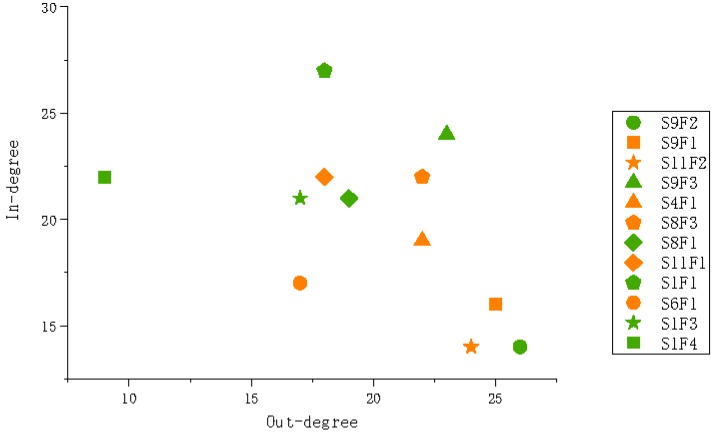
Dominance of the influencing factor based on in-degree centrality and out-degree centrality.

Although the cohesion value indicates that the network is relatively complex, ranking all the influencing factors would identify and demonstrate which of the factors is worth more attention, thus prioritizing the strategies for green building adoption and promotion activities. Figure 3 displays the in-degree and out-degree of the influencing factors introduced in Equations (3) and (4), respectively. These two indicators expressed the characteristics and effects of the influencing factor nodes from different perspectives.

Here, twelve factors are selected in the diagram because they either showed a high degree of impact from others or had a large number of direct successors. In Figure 3, S9F2 “force of supervision in the process of construction” has the highest out-degree of 26. This result indicates that the level of government supervision can affect most factors and related stakeholders. S9F1 “promulgate laws and regulation of green building” with an out-degree of 16 and an in-degree 9 can affect other influencing factors generally but has a relatively low direct impact from the others. In the diagram, S1F4 “incremental cost of green building” is a special case. Although having an out-degree of 18, S1F4 can also be affected readily by an immediate predecessor with an in-degree of 27. In practice, although some incentive policies have been implemented, the developers, as the primary providers of funding, will often change the investment strategy to ensure that the increase in green building costs is within the expected range. A majority of influencing factors are ordinary nodes in the network, while eight of the influencing factors more easily accept influences from their neighbours than direct outcome impacts. These influencing factors are listed in Table 3. The existence of these influencing factors will have a minimal effect on the complexity of the network.

The indicators of betweenness centrality express the degree to which a factor or an interrelation can control the impacts passing through it, i.e., the ability to control impact. The top ten influencing factors and the interactions ranked by betweenness centrality are shown in Table 4. S8F1 “cognition about green buildings” has the highest betweenness centrality in the network. On the one hand, users’ perceptions about green buildings come from the activities of other stakeholders. On the other hand, users’ perceptions also influence other stakeholders to promote the development of green buildings. Thus, “understanding green buildings” acts as hubs connecting many pairs of influencing factors. Ten of the most important links and the influencing factors are associated with them are displayed in Table 4. The source factors of the links in Table 4 should be treated with caution. A comparison of the influencing factors in Table 4 and Figure 3 shows that two influencing factors, S6F1 “experience of manage green building” and S11R1 “media and public recognition and spread of information on green buildings” does not directly have a high impact and still plays an important role in network connectivity.

**Figure 4:**
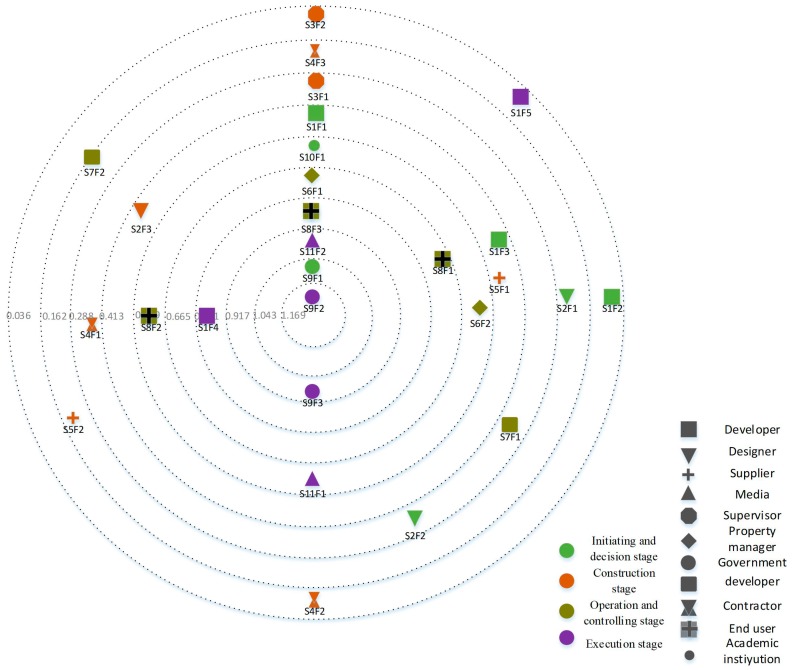
Influencing factors in the status centrality map.

Due to a space limitation, only the top ten influencing factors and their out-status centrality scores are listed in Table 5. The status centrality map, including all influencing factors, is shown in Figure 4. In this analysis, the most significant influencing factors are S9F1 “promulgate laws and regulations for green buildings” and S9F2 “level of supervision in the process of construction”. In Figure 4, the influencing factor impacts decrease with the distance between the influencing factor (node) and the centre of the circle. The legends show that different shapes represent the stakeholder groups, while colours represent the influencing factor categories.

Although most of the influencing factors in Table 4 have already been identified according to degree and betweenness centrality, one influencing factor, S1F1 “green target location”, is deemed critical when considering global interactions in the network. Therefore, developers should clear green targets and enhance communication activities with other stakeholders.

## 5. Discussion

Green buildings have been perceived as a major innovation in the construction industry that can accomplish the synergy of environmental protection, economic development and social responsibility. This study identifies some critical factors influencing the successful and widespread adoption of green buildings in China. In what follows, we discuss the results obtained from Section 4.

(1) The survey results indicate that “level of supervision in the process of construction” is perceived to be an important influencing factor in the social network. While Chinese green building-related laws and standard systems have increasingly captured the attention of the construction industry in recent years, the implementation of green buildings is not as planned [53]. Many stakeholders, such as contractors, clients and suppliers, maintain unwilling attitudes towards the adoption of green buildings. By nature, it is true that most stakeholders are accustomed to traditional construction and will not voluntarily change. Thus, to achieve successful and widespread adoption of green buildings in China, it is necessary to change the attitude of existing stakeholders about these projects. As the main advocate of green buildings, the supervision and management of the government directly affect the entire life stage of a project and the stakeholders involved. These issues may explain why level of supervision in the process of construction is considered a critical influencing factor that inhibits the adoption of green buildings in China. These effects are also important reasons that green buildings fail to reach design standard values during actual operation.
(2) As a critical influencing factor to implementing green buildings in China, “property experience and attitude towards green building properties” has attracted the attention of scholars. Many scholars found that more than 80% of energy consumption occurs during the actual occupancy operational stage rather than during the construction stage, which indicates that the performance of green buildings largely depends on the management level of the property in the occupancy operational stage [51,52]. Therefore, managing the property during the operational stage of a green building can affect other influencing factors and related stakeholders, such as clients, designers and contractors, involved in the life cycle of the project.
(3) As expected, the incremental cost of green building is an important influencing factor in the social network that has a high in-degree and high out-degree. Green buildings are widely considered as requiring additional costs for either design or green technologies and materials. In the life cycle of a green building, almost every stakeholder shows concern about the cost increases at first [32]. In particular, some undeveloped cities in China, the cost increments directly affect other factors. Thus, higher costs will not only reduce the positive attitude of most stakeholders to green buildings but also increase the ratio of users who prefer to buy traditional buildings at a lower price. This scenario is also an important cause of the uneven development of green buildings between different regions. In addition, the influencing factors include the experience of stakeholders in green building, target positioning of green building, rationality of design and other factors that always increase the cost of green buildings during project implementation. Thus, the cost increase for green buildings is also easily affected by other influencing factors.
(4) A designer’s and contractor’s roles are not as important as perceived and can be found in the results obtained from the social network. This finding supports previous research that demonstrates that with the continuous improvement of green building standards and the increasing number of green buildings, many contractors and designers have accumulated considerable experience [12]. The development of various computer software has also increased the convenience of acquiring information and improving technology. Thus, these influencing factors do not occupy a core position in the network. Similar to designers and constructors, the promotion of green building materials by suppliers is not as important as perceived. In the market, new energy materials and technical equipment have already occupied a large share. Many developers also using various energy-saving and environmental protection systems, such as solar panels and rainwater recycling, in new buildings [49]. Therefore, the promotion of new energy and new materials in the market are not considered a critical influencing factor in the social network.
(5) An interesting finding is that end users and public awareness of green buildings have become very important in the social network. Currently, the knowledge and understanding of green building by end users and the public need to be further promoted [24]. Although an increasing number of residents recognized that environmental pollution is a serious issue, they often ranked this issue as a social one related to government and business. Especially in the current market environment, very few people are concerned about whether a house purchased is a green building [19]. In addition, due to the lack of awareness about green buildings, it is difficult for end users to achieve energy savings and reduce costs during the occupancy operational stage. This scenario leads the public to think that green buildings are the realization of environmental protection at high costs and that many stakeholders prefer traditional construction.

## 6. Conclusions

This paper leverages the collective knowledge of influencing factors and stakeholders from a network perspective to achieve better management in the life cycles of green buildings. Several important findings stem from this study: (1) The level of government supervision in the process of construction plays a more important role during the life cycle of green building compared to promulgate laws and regulations. (2) Property management experience and attitude in the green building operational stage are considered more significant to determining the various functions of green buildings, as expected. (3) As an important factor, the increasing costs of green buildings affect other factors and related stakeholders such as clients, designers, contractors and end users involved in the life cycle of the project. (4) Some influencing factors, such as the experience of designers and contractors and the materials of green buildings, are not as important as perceived. (5) End users and public awareness of green buildings have become very important in the social network. Although these findings need to be verified with more in-depth investigations and case studies, the outcomes of this study can facilitate decision-makers from both private and public sectors who are involved in green building.

Based on the analytical results, some suggestions for promoting the development of green buildings are made as follows: First, the government’s responsibility should not only improve the green building legal system but also adopt effective evaluation standards to carefully supervise the green building life cycle. Second, property managers should have extensive experience with green buildings or pass professional qualification training, such as energy usage simulation software. Third, the government should be more responsive to the sustainable trend in the construction industry by implementing some coupling regulations with incentives to increase the value and lower operating costs of green buildings. Expedited permits and tax exemptions are two potential widely used policies to encourage green building. Finally, end users and public awareness of green buildings can be promoted by policy guidance and social education. It is essential to provide a clear definition of genuine green buildings and make the public clearly aware the necessity of green building development.

There may be two research limitations in this study. First, it is worth noting that the weights of different respondents are ignored in the survey. This limitation may affect the accuracy of the research result. Therefore, the knowledge and contribution of respondents should be considered in the future research. Second, although this study has identified the crucial influencing factors by SNA, the path analysis factors and the influence level in different factors are not clear. Both of them will be considered in our future research.

## Figures and Tables

**Table 1:** Number of green buildings in China.

	Year	2008	2009	2010	2011	2012	2013	2014	2015	Total
Category										
1. Total		10	20	82	241	386	509	916	1091	3255
2. Rating Level										
- Commercial Buildings										
- One-star		1	3	4	28	67	86	191	258	638
- Two-star		2	4	19	37	60	71	156	220	569
- Three-star		3	9	14	35	61	65	121	148	456
- Residential Buildings										
- One-star		3	1	10	48	74	93	190	221	640
- Two-star		0	2	25	50	93	161	185	199	715
- Three-star		1	1	10	43	31	33	73	45	237
3.Rating Period										
- Commercial Buildings										
- Design		6	14	31	93	167	194	434	608	1547
- Operation		0	2	6	7	21	28	34	18	116
- Residential Buildings										
- Design		4	4	43	135	194	265	431	444	1520
- Operation		0	0	2	6	4	22	17	21	72

**Table 2:** The stakeholders and related influencing factors.

Sources	Factor of Categories	Influencing Factor	Project Life-Cycle	Stakeholder
[17,18,19]	S1F1	Green target location	Initiating and decision stage	Developer
	S1F2	Analyse extent of the effect of green target on project execution		
	S1F3	Positive attitude to green buildings		
	S1F4	Incremental cost of green buildings	Execution stage	
	S1F5	Experience managing green buildings		
[18,49]	S2F1	Experience designing green buildings	Design substage	Designer
	S2F2	Analyse extent of the project		
	S2F3	Rationality of design plan	Construction substage	
[50]	S3F1	Experience managing green buildings	Construction substage	Supervisor
	S3F2	Working attitude towards green buildings		
[26,31,32]	S4F1	Construction experience with green buildings	Construction substage	Contractor
	S4F2	Capability level related to green buildings		
	S4F3	Ability to manage green buildings in the process of construction		
[20,27]	S5F1	New energy and new materials spread in the market	Construction substage	Supplier
	S5F2	Supply of quality goods on time		
[51,52]	S6F1	Experience managing green buildings	Controlling substage	Property manager
	S6F2	Working attitude towards green building		
[23]	S7F1	Experience evaluating green buildings	Operation substage	Assessor/certifier
	S7F2	Equity in the process of evaluating		
[18,51]	S8F1	Understanding about green buildings	Operation substage	End user/client
	S8F2	Protecting green buildings during use	Controlling substage	
	S8F3	Tendency degree of purchasing green buildings	Operation substage	
[17,23,53]	S9F1	Promulgating laws and regulation of green buildings	Initiating and decision stage	Government
	S9F2	Level of supervision in the process of construction	Execution stage	
	S9F3	Recognizing and promoting new materials and new technology		
[50]	S10F1	Studies of green buildings	Initiating and decision stage	Academic institution
[24,25,29]	S11F1	Recognizing and spreading information on green buildings		Media and public
	S11F2	Supervision attitude in green building	Execution stage	

**Table 3:** Receivers in the influencing factor network.

Factor ID	Influence Factor	Associated Stakeholder
S1F2	Analyze extent about the effect of green target to project execute	Developer
S1F3	The positive attitude to green building	Developer
S2F1	Experience of design green building	Designer
S2F2	Analyze extent about the project	Designer
S2F3	The rationality of design plan	Designer
S3F1	Experience of manage green building	Supervisor
S4F2	The capability level about green building	Contractor
S8F2	Protect the green building during use	End user

**Table 4:** The key factor related to betweenness centrality.

Rank	Factor ID	Node Betweenness Centrality	Link ID	Link Betweenness Centrality
1	S8F3	26.217	S8F3→S3F2	4.981
2	S9F2	19.013	S4F2→S11F2	4.955
3	S1F1	16.707	S1F4→S6F2	4.852
4	S9F1	15.344	S1F4→S1F3	4.420
5	S8F1	14.326	S6F2→S9F2	4.025
6	S4F1	14.169	S4F1→S3F2	3.693
7	S2F3	13.727	S9F2→S3F2	3.493
8	S6F1	13.366	S11F1→S9F2	3.278
9	S1F3	13.099	S11F1→S7F2	3.154
10	S11F1	12.825	S6F1→S9F2	3.126

**Table 5:** The key factor related to out-status centrality.

Rank	Influence Factor	Out-Status Centrality
1	S1F4	1.361
2	S1F1	1.360
3	S8F3	1.349
4	S9F2	1.349
5	S9F1	1.197
6	S11F1	1.096
7	S8F1	0.970
8	S2F2	0.843
9	S6F1	0.838
10	S1F2	0.776

## References

[1] Sina Finance (2015). China’s Added New Buildings Exceeded 2 Billion Square Meters (Annually), Accounting for More Than Half the Word’s Amount. http://finance.sina.com.cn/money/roll/20150327/144521827153.shtml.

[2] World Business Council for Sustainable Development (2007). Energy Efficiency in Buildings, Business Realities and Opportunities.

[3] Berardi U. (2017). A cross-country comparison of the building energy consumptions and heir trends. Resour. Conserv. Recycl..

[4] Wu X., Peng B., Lin B. (2017). A Dynamic Life Cycle Carbon Emission Assessment on Green and Non-Green Buildings in China. Energy Build..

[5] Shen L., Yan H., Fan H., Wu Y., Zhang Y. (2017). An integrated system of text mining technique and case-based reasoning (TM-CBR) for supporting green building design. Build. Environ..

[6] USEPA Definition of Green Building. https://archive.epa.gov/greenbuilding/web/html/about.

[7] Zhao D.X., He B.J., Johnson C. (2015). Social problems of green buildings: From the humanistic needs to social acceptance. Renew. Sustain. Energy Rev..

[8] Iheanyichukwu J.O., Godwin U.A., Shahril A.R. (2018). Modelling the effects of green building incentives and green building skills on supply factors affecting green commercial property investment. Renew. Sustain. Energy Rev..

[9] Lang S. (2004). Progress in energy-efficiency standards for residential buildings in China. Energy Build..

[10] Ministry of Housing and Urban-Rural Development of the People’s Republic of China (2014). Evaluation Standard for Green Building; GB/T 50378-2014.

[11] Li J., Wang X. (2012). Energy and climate policy in China’s twelfth five-year plan: A paradigm shift. Energy Policy.

[12] Li Z., Wu J., Liu H. (2018). Polices to enhance the drivers of green housing development in China. Energy Policy.

[13] Zou Y.H., Zhao W., Zhong R. (2017). The spatial distribution of green buildings in China: Regional imbalance, economic fundamentals, and policy incentives. Appl. Geogr..

[14] Ye L., Cheng Z., Wang Q., Lin W., Ren F. (2013). Overview on green building label in China. Renew. Energy.

[15] Darko A., Zhang C., Chan A.P. (2017). Drivers for green building: A review of empirical studies. Habitat Int..

[16] Sharma M. (2018). Development of a ‘Green Building Sustainability Model’ for Green Buildings in India. J. Clean Prod..

[17] Zhang X., Li Y.S., Wu Y. (2011). Green strategy for gaining competitive advantage in housing development: A China study. J. Clean Prod..

[18] Yau Y. (2012). Eco-labels and willingness-to-pay: A Hong Kong study. Smart Sustain. Built Environ..

[19] Hu H., Geertman S., Hooimeijer P. (2014). The willingness to pay for green apartments: The case of Nanjing, China. Urban Stud..

[20] Lam P.T.I., Chan E.H.W., Poon C.S. (2010). Factors affecting the implementation of green specifications in construction. J. Environ. Manag..

[21] Qian S., Jian Z., George Z. (2012). Exploring the management of sustainable construction at the programme level: A Chinese case study. Constr. Manag. Econ..

[22] Shen L.Y., Tam V.W.Y., Tam L. (2010). Project feasibility study: The key to successful implementation of sustainable and socially responsible construction management practice. J. Clean Prod..

[23] Lee W.L., Chen H. (2008). Benchmarking Hong Kong and China energy codes for residential buildings. Energy Build..

[24] Li Y., Yang L., He B. (2014). Green building in China: Needs great promotion. Sustain. Cities Soc..

[25] Bai L., Wang H., Huang N. (2018). An Environmental Management Maturity Model of Construction Programs Using the AHP-Entropy Approach. Int. J. Environ. Res. Public Health.

[26] Shi Q., Zuo J., Huang R. (2013). Identifying the critical factors for green construction—An empirical study in China. Habitat Int..

[27] Zhu Y., Lin B. (2004). Sustainable housing and urban construction in China. Energy Build..

[28] Hoffman A.J., Henn R. (2008). Overcoming the social and psychological barriers to green building. Organ. Environ..

[29] Cole R.J., Brown Z. (2010). Building human agency: A timely manifesto. Build. Res. Inf..

[30] Leman A., Stevension F., Bordass B. (2010). Building evaluation: Practice and principles. Build. Res. Inf..

[31] Qi G.Y., Shen L.Y., Zeng S.X., Jorge O.J. (2010). The drivers for contractors’ green innovation: An industry perspective. J. Clean. Prod..

[32] Liu J.Y., Low S.P., He X. (2012). Green practices in the Chinese building industry: Drivers and impediments. J. Technol. Manag. China.

[33] Berry S., Davidson K., Saman W. (2013). The impact of niche green building developments in transforming the building sector: The case study of Lochiel Park. Energy Policy.

[34] Moreno J.L. (1960). The Sociometry Reader III.

[35] Hagedoorn J. (1996). Trends and patterns in strategic technology partnering since the early seventies. Rev. Ind. Organ..

[36] Park S., Lee S.J., Jun S. (2015). A network analysis model for selecting sustainable technology. Sustainability.

[37] Ghali M.R., Frayret J.M., Robert J.M. (2016). Green social networking: Concept and potential applications to initiate industrial synergies. J. Clean. Prod..

[38] Wasserman S., Galaskiewicz J. (1960). Advances in Social Network Analysis: Research in the Social and Behavioral Sciences.

[39] Scott J. (2000). Social Network Analysis: A Handbook.

[40] Yang R.J., Zou P.X. (2014). Stakeholder-associated risks and their interactions in complex green building projects: A social network model. Build. Environ..

[41] Steward D. (1981). The design structure matrix: A method for managing the design of complex systems. IEEE Trans. Eng. Manag..

[42] Fang C., Marle F., Zio E. (2005). Network theory-based analysis of risk interactions in large engineering projects. Reliab. Eng. Syst. Saf..

[43] Chinowsky P., Diekmann J., Galotti V. (2008). Social network model of construction. J. Constr. Eng. Manag..

[44] Parise S. (2007). Knowledge management and human resource development: An application in social network analysis methods. Adv. Dev. Hum. Resour..

[45] Loosemore M. (1998). Social network analysis: Using a quantitative tool within an interpretative context to explore the management of construction crises. Eng. Constr. Arch. Manag..

[46] Newman M.E. (2001). Scientific collaboration networks. II. Shortest paths, weighted networks, and centrality. Phys. Rev..

[47] Katz L. (1953). A new status index derived from sociometric data analysis. Psychometrika.

[48] Yang R.J., Zou P.X., Wang J.Y. (2016). Modelling stakeholder-associated risk networks in green building projects. Int. J. Proj. Manag..

[49] Chan E.H., Qian Q.K., Lam P.T. (2009). The market for green building in developed Asian cities-the perspectives of building designers. Energy Policy.

[50] Li H., Ng S.T., Skitmore M. (2018). Stakeholder impact analysis during post-occupancy evaluation of green buildings—A Chinese context. Build. Environ..

[51] Liang X., Hong T., Shen G.Q. (2016). Occupancy data analytics and prediction: A case study. Build. Environ..

[52] Liang X., Hong T., Shen G.Q. (2016). A game theory based analysis of decision making for green retrofit under different occupancy types. J. Clean. Prod..

[53] Shi Q., Lai X., Xie X. (2014). Assessment of green building policies—A fuzzy impact matrix approach. Renew. Sustain. Energy Rev..

